# 

**Published:** 1916-08-26

**Authors:** 


### CONTENTS.

| | PAGE |
|---|---|
| **Some Hospital Statistics: Their Use and Value** ... ... ... ... ... ... ... | 481 |
| **Hospitals and the Spirit Tax** ... ... ... | 482 |
| **Hospital and Institutional News** ... ... ... | 483 |
| Hospitals and the National Relief Fund—A Wonderful Week for Hospital Legacies—£165,000 for Scottish Institutions—A Picture Gallery in a Hospital—Relations and the Whereabouts of Wounded Soldiers—A Startling Suggestion—The Importation of Infantile Paralysis—On Tour Among the Wounded—Economy in Hospital Gardens—A Record Admission—An Unusual Case—Entertaining Wounded at English Ports—Hospital Enterprise in Aberdeen—How to Recoup an Annual Subscription—A Working Man's Convalescent Home—The Hospital Saturday Fund in Leicester—Lodgings in the Workhouse—An Innovation in Scotland—A Successful Appeal—The Mutability of the Hospital Staff—The Winchester Council and the Hampshire County Hospital—The Insurance of Hospital Buildings—Typhus Fever in Glasgow—A New Hospital at Thurgarton—London's Sanatorium Deficiencies—The Liberty of the Tuberculous Subject—A Deadlock in Pembrokeshire—Infant Welfare in Denbighshire—The C.E.T.S. and the Army—Cocaine for Unregistered Dentists—Race Suicide at Derby—This Week's Drug Market. | |
| **The State Treatment of Venereal Diseases** ... | 489 |
| The New Regulations. | |
| **Increased Income for Charitable Institutions** | 490 |
| The Fulfilment of a Patriotic Duty. | |
| **Out-Patients and the War** ... ... ... ... | 491 |
| The Experiences of Westminster Hospital. | |
| **A New Use for Hospital Gardens** ... ... | 492 |
| The Cultivation of Medicinal Herbs. | |
| **The Matrons' and Sisters' Department** ... | 493 |
| Mental Nurses in Military Hospitals. | |
| Round the Hospitals. | |
| Follow In-Patients Home—Miss Haughton's Yeoman Service—British Red Cross in Italy—The Nightingale Fund: Miss Lloyd Still's Success—Two Aspects of V.A.D. Work. | |
| **The King George Hospital** ... ... ... ... ... | 495 |
| Some Ward Sisters and Staff Nurses Protest. | |
| **Passing Events and Topics**... ... ... ... ... | 496 |
| **The Editor's Letter-Box** ... ... ... ... ... | 497 |
| Surbiton Cottage Hospital and the Sunday Fund Award—Nurses' Indoor Uniform in the Streets: Unhygienic and Unprofessional—Mental Nurses and War Hospital Work. | |
| **Parliament and the Profession** ... ... ... ... | 498 |
| **Matrons' and Sisters' Appointments** ... ... | vi |
| **Gifts to Hospitals** ... ... ... ... ... ... | vi |
| **The College of Nursing, Limited** ... ... | vi |
| **The Institutional Worker** ... ... (Suppl.) | 1 |

